# Modification of the Mycotoxin Deoxynivalenol Using Microorganisms Isolated from Environmental Samples

**DOI:** 10.3390/toxins9040141

**Published:** 2017-04-15

**Authors:** Nina M. Wilson, Nicole McMaster, Dash Gantulga, Cara Soyars, Susan P. McCormick, Ken Knott, Ryan S. Senger, David G. Schmale

**Affiliations:** 1 Department of Plant Pathology, Physiology, and Weed Science, Virginia Tech, Blacksburg, VA 24061, USA; nina09@vt.edu (N.M.W.); niki@vt.edu (N.M.); gantulga@vt.edu (D.G.); 2 Biology Department, The University of North Carolina at Chapel Hill, Chapel Hill, NC 27599, USA; csoyars@live.unc.edu; 3 USDA-ARS, Mycotoxin Prevention and Applied Microbiology, Peoria, IL 61604, USA; susan.mccormick@ars.usda.gov; 4 Department of Chemistry, Virginia Tech, Blacksburg, VA 24061, USA; kknott@vt.edu; 5 Department of Biological Systems Engineering, Virginia Tech, Blacksburg, VA 24061, USA; senger@vt.edu; 6 Department of Chemical Engineering, Virginia Tech, Blacksburg, VA 24061, USA

**Keywords:** mycotoxin, trichothecene, deoxynivalenol, bioprospecting, detoxification, *Fusarium*

## Abstract

The trichothecene mycotoxin deoxynivalenol (DON) is a common contaminant of wheat, barley, and maize. New strategies are needed to reduce or eliminate DON in feed and food products. Microorganisms from plant and soil samples collected in Blacksburg, VA, USA, were screened by incubation in a mineral salt media containing 100 μg/mL DON and analysis by gas chromatography mass spectrometry (GC/MS). Two mixed cultures derived from soil samples consistently decreased DON levels in assays using DON as the sole carbon source. Nuclear magnetic resonance (NMR) analysis indicated that 3-keto-4-deoxynivalenol was the major by-product of DON. Via 16S rRNA sequencing, these mixed cultures, including mostly members of the genera *Acinetobacter*, *Leadbetterella*, and *Gemmata*, were revealed. Incubation of one of these mixed cultures with wheat samples naturally contaminated with 7.1 μg/mL DON indicated nearly complete conversion of DON to the less toxic 3-epimer-DON (3-epi-DON). Our work extends previous studies that have demonstrated the potential for bioprospecting for microorganisms from the environment to remediate or modify mycotoxins for commercial applications, such as the reduction of mycotoxins in fuel ethanol co-products.

## 1. Introduction

Mycotoxins are toxic secondary metabolites produced by fungi that are a threat to the health of humans and domestic animals [1]. This diverse class of compounds can contaminate commercial foods (e.g., wheat, maize, peanuts, cottonseed, and coffee) and animal feedstocks. Mycotoxins can be harmful even at small concentrations, creating significant food safety concerns [1,2]. The Food and Agriculture Organization estimated that approximately 1 billion metric tons of food is lost each year due to mycotoxin contamination [3]. Economic losses include yield loss from mycotoxin contamination [4], reduced value of crops [4], loss of animal productivity from health issues related to mycotoxin consumption [5], and even animal death [6,7].

The trichothecenes are a major class of mycotoxins containing over 150 toxic compounds and are toxic inhibitors of protein synthesis [8,9]. Trichothecenes are produced by several different fungi in the genus *Fusarium* [9,10]. One of the most economically important trichothecenes is deoxynivalenol (DON), which contaminates wheat, barley, and maize worldwide [11]. DON causes feed refusal, skin disorder, diarrhea, reduced growth, and vomiting in domestic animals [12]. Depending on the dose and exposure time of DON, there is also evidence that DON acts as an immunosuppressive [1]. It is among the most closely monitored mycotoxins in the US, and DON contaminations have resulted in estimated annual losses of up to $1.6 billion [13].

While there is structural variety, all trichothecenes share a core structure that includes the C-12,13 epoxide that is important to toxicity and protein inhibition [14,15]. DON is a type B trichothecene characterized by the presence of a keto group on C-8 [16]. There are mechanisms the fungus *Fusarium* implements during the biosynthesis of DON to alter the structure, making it less toxic, e.g., acetylating the C-3 position [16].

Microbial detoxification of mycotoxins has previously been reported [17,18]. Fuchs et al. [19] were able to isolate an anaerobic eubacterium that converted DON to de-epoxy-DON. A few years later, Völkl and colleagues [20] reported that a mixed culture of organisms from soil samples converted DON to 3-keto-4-deoxynivalenol (3-keto-DON), but they were unable to identify the causal microorganisms responsible for the modification. The product 3-keto-DON is approximately 90% less toxic than DON, and represents a suitable detoxified product [21]. Shima et al. [21] discovered a single organism in aerobic conditions from an environmental sample that converted DON into 3-keto-DON, and He et al. [22,23] isolated an aerobic organism, from the genus *Devosia*, converting DON to 3-epimer-DON (3-epi-DON). Ikunaga et al. [24] identified a bacterium from the genus *Nocardioides* that converts DON to 3-epi-DON. Recently, He et al. [25] discovered an aerobic culture of microorganisms converting DON to de-epoxy-DON. The current study extends these prior investigations to a series of studies to isolate additional microorganisms from the environment that modify and remediate DON. While others have shown that soil bacteria can detoxify DON, the functional enzyme(s) responsible for conversion to 3-keto-DON remains elusive. Once the enzymatic mechanism(s) and genetic element(s) responsible are identified, yeast can be engineered to remediate DON during a fermentation process involving mycotoxin-contaminated feedstocks.

Based on previous work [21,22,23,24,25], we hypothesized that mixed cultures of microorganisms isolated from natural soil environments incubated with a mineral salt media using 100 μg/mL DON as the sole carbon source will detoxify DON. The specific objectives of this research were as follows: (1) identify microbes isolated from plant and soil samples taken in Blacksburg, VA, that modify DON; (2) characterize DON metabolites using thin layer chromatography (TLC), gas chromatography mass spectrometry (GC/MS), and nuclear magnetic resonance (NMR); (3) identify bacterial components of mixed cultures with DON modification activity; and (4) determine if these microorganisms can modify DON in naturally contaminated wheat samples. Our work extends previous studies that have demonstrated the potential for bioprospecting for microbes that modify toxic secondary metabolites from grains and/or grain products, such as the reduction of mycotoxins in fuel ethanol co-products.

## 2. Results

### 2.1. Selection of Microbes in the Presence of High Concentrations of DON

An initial screen of 11 plant and soil environmental samples incubated in mineral media containing 100 μg/mL DON as the sole carbon source identified five cultures in which no DON remained after 7 days. These five mixed culture samples that eliminated DON from the culture media (below the limit of quantification (<LOQ), which was 0.2 μg/mL) came from soil samples taken from a landscape plot, vineyard, and peach orchard and from plant samples taken in a small grain field and a vineyard. With further subculturing, three mixed culture samples had decreased DON levels in the culture media (Table S1), all of which were derived from the landscape plot. Only two samples from the landscape plot, Mixed Cultures 1 and 2 (Figure S1), consistently removed/modified DON in the culture media. Further assays with Mixed Cultures 1 and 2 (Table 1) suggested that the glycerol stocks were heterogeneous and likely contained mixtures of culturable and unculturable microbes; four sample replicates from Mixed Cultures 1 and 2 did not perform the same and had varying amounts of DON modification based on percentage of DON modified in each culture (Table 1). Mixed Culture 3 replicates did not modify DON and thus were not studied further.

### 2.2. Isolation of Individual DON Modifying Microbes

There were two pure cultures, Pure Cultures 1 and 2 from Table S1, of bacteria that were initially associated with decreased levels of DON within culture media. Pure Culture 1 originated from the small grain field, and Pure Culture 2 originated from the landscape plot. Via 16S rRNA sequencing, it was revealed that Pure Culture 1 was from the genus *Achromobacter*, and the Pure Culture 2 was from the genus *Pseudomonas*. However, additional DON assays indicated that both Pure Cultures 1 and 2 did not consistently modify DON (data not shown). The two pure cultures were inconsistent in their ability to eliminate DON from culture media, although preparation and culture conditions remained the same.

### 2.3. Identification of Mixed Cultures Using 16S Ribosomal Sequencing

**Figure 1:**
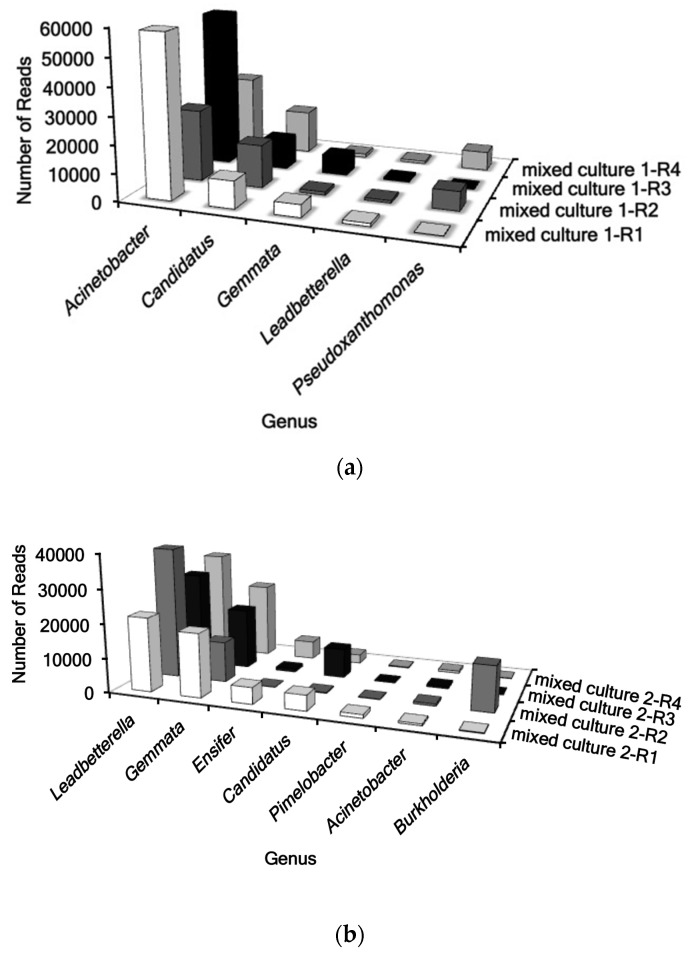
16S rRNA sequencing data for within sample repetitions of (**a**) Mixed Culture 1 and (**b**) Mixed Culture 2. Each plot graph illustrates the major genera represented in each sample in accordance with number of reads resolved from sequencing. In (**a**) Mixed Culture 1-R1 was the only culture that was able to modify DON from the culture media. In (**b**) Mixed Culture 2-R1, Mixed Culture 2-R3, and Mixed Culture 2-R4 were able to modify DON from the culture media.

Sequencing of 16S of Mixed Cultures 1 and 2, which consistently modified DON, indicated that they contained mostly members of the genera *Acinetobacter*, *Leadbetterella*, and *Gemmata* (Figure 1). Mixed Culture 1 consisted of members of the genera *Acinetobacter*, while Mixed Culture 2 was composed mostly of the genera *Leadbetterella*, and *Gemmata*. Mixed Culture 1-R1 was the only culture able to modify DON in culture media (Figure 1a). Mixed Culture 1-R1 was composed mostly of the genera *Acinetobacter* and *Candidatus*. Mixed Culture 2-R1, Mixed Culture 2-R3, and Mixed Culture 2-R4 modified DON (Figure 1b). Mixed Culture 2-R2 was unable to modify DON in culture media and was the only culture that contained a large amount of *Burkholderia*.

### 2.4. Thin Layer Chromatography to Identify DON Derivatives

**Figure 2:**
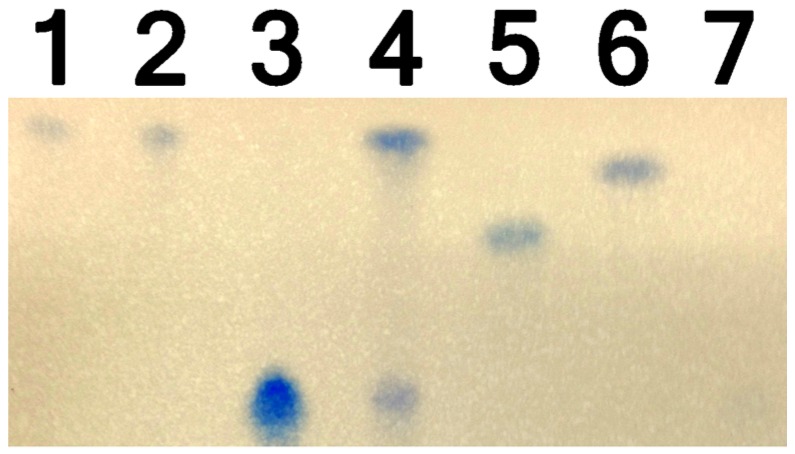
TLC analysis of DON by-products and controls. Lane 1: Mixed Culture 1; Lane 2: Mixed Culture 2; Lane 3: DON; Lane 4: 3-keto-DON and DON mixture; Lane 5: 15-A-DON; Lane 6: 3-A-DON: and Lane 7: De-epoxy-DON.

TLC analysis of extracts of Mixed Cultures 1 and 2 showed a DON by-product that was less polar than DON, 15-ADON, and 3-A-DON (Figure 2). Mixed Cultures 1 and 2 by-products showed similar properties to 3-keto-DON, which is nonpolar and migrated further up the TLC plate. Mixed Cultures 1 and 2 by-products were also dissimilar to the acetylated versions of DON, 15-A-DON, and 3-A-DON. The product turned blue with NBP/TEPA, indicating that it contained an epoxide, and had a similar Rf (retention factor) as 3-keto DON.

### 2.5. Nuclear Magnetic Resonance to Identify Structure of DON Derivatives

NMR analysis showed that both the DON by-product in both Mixed Culture 1 and Mixed Culture 2 was 3-keto-deoxynivalenol (3-keto-DON) (Table S2). The 3-keto-DON proton closely resembled that reported by Shima et al. [21].

### 2.6. DON Assays with Mixed Cultures with Naturally Contaminated Wheat Samples

**Figure 3:**
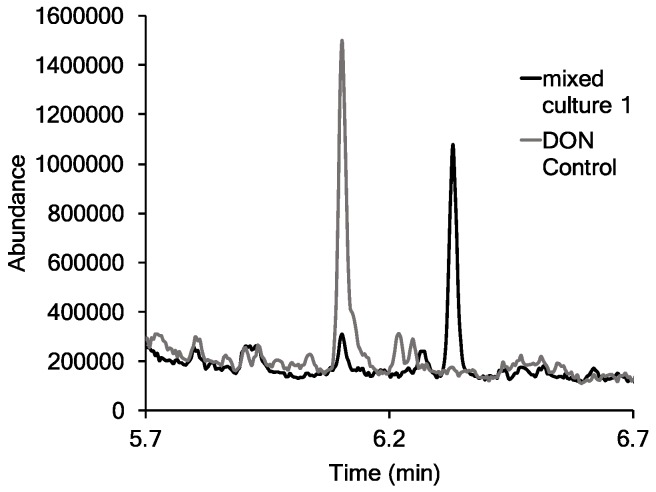
GC/MS chromatograph of DON derivatives in scan operating mode from Sample ID 2 in Table 2 (Mixed Culture 1, Wheat Sample #13w-7, 7.1 μg/mL DON). DON (grey line; retention time of 6.12 min) was modified to 3-epi-DON (black line; retention time of 6.33 min).

DON assays using Wheat Sample #13w-7 (41.0 μg/mL) did not show any DON reduction with Mixed Cultures 1 or 2. DON reduction was observed with Wheat Sample #13-v193 (7.1 μg/mL DON). In particular, two samples from Mixed Culture 1 (Sample ID 2 and Sample ID 3 in Table 2) showed nearly complete DON reduction compared to the control. Figure 3 shows a GC/MS chromatogram overlay of the DON control and Sample ID 2 from Mixed Culture 1, where significant DON reduction was observed. The DON peak had a retention time of 6.12 min, and the new peak at 6.33 min is postulated to be 3-epi-DON based on molecular weight and fragmentation. According to He et al. [22], 3-epi-DON is significantly less toxic than DON. However, Ikunaga et al. [24] suggest that 3-epi-DON may still be just as toxic as DON since the epoxide ring is still present. DON and the postulated 3-epi-DON were detected in SIM mode with a target ion with a mass/charge ratio of 512.3 and had reference ions at 422.4 and 497.3.

## 3. Discussion

New strategies are needed to reduce or eliminate DON in feed and food products. DON degrading activity restricted to anaerobic organisms limits the potential use of these microorganisms for industrial purposes as feed additives. Aerobic organisms pose their own problems, as many valuable organisms are unculturable in the lab. Even though culturing aerobic organisms from the environment can be tedious, Shima et al. [21] discovered Strain E3-39, which can convert DON into 3-keto-DON under aerobic conditions, and He et al. [25] discovered a microbial culture that converted DON to de-epoxy-DON. Here, we extend these prior studies by isolating mixed microbial cultures from the environment that modify DON, characterizing their DON derivatives, and characterizing the microorganisms present in cultures that modify DON.

Two mixed cultures that consistently decreased DON in cultures, in which DON was the sole carbon source in a minimal medium, were identified. From these mixed cultures, we were unable to isolate a pure culture that modified DON consistently in culture media. Several bacteria and fungal colonies were initially selected and screened for DON modification, but only two bacteria eliminated DON from cultures containing DON as the sole carbon source. These two bacteria, an *Achromobacter* and *Pseudomonas* species, were not consistent in eliminating DON from cultures. This inconsistency could be due to the cultures being stored in glycerol stocks at −80 °C, since the cold temperatures may have affected their ability to modify DON. Völkl et al. [20] also were unable to identify a pure organism from the mixed culture (D107) that consistently modified DON. Isolating pure cultures remains a challenge, as multiple microorganisms could be responsible for the metabolism or conversion of DON.

According to Shima et al. [19], 3-keto-DON is significantly less toxic than DON. Proton data from Mixed Cultures 1 and 2 were similar to 3-keto-DON reported by Shima et al. [21]. There is a discrepancy in the literature regarding proton data for 3-keto-DON reported by Völkl et al. [20]; the authors appear to have inadvertently switched some of the proton data for DON with the proton data for 3-keto-DON.

Results from 16S rRNA sequencing of Mixed Cultures 1 and 2, which consistently modified DON, indicated that they contained mostly members of the genera *Acinetobacter*, *Leadbetterella*, and *Gemmata*. To our knowledge, these genera have not been reported previously to modify DON in culture. Strains of *Acinetobacter* have been associated with the modification of ochratoxin A [26]. He et al. reported *Pseudomonas* and *Achromobacter* genera in their microbial culture that converted DON to de-epoxy-DON; the two pure cultures we isolated that demonstrated activity in DON cultures could have lost functionality during storage in glycerol stocks at −80 °C. Isolating DON modifying microbes is difficult, in part due to growth and function restrictions since some microbes may be inhibited by others [27]. Several microorganisms are likely responsible for the conversion of DON to 3-keto-DON, and with additional testing and analysis it may be possible to isolate the specific bacteria responsible for DON modification.

DON was nearly eliminated in two naturally contaminated samples of wheat (7.1 μg/mL DON) inoculated with Mixed Culture 1. GC/MS scans of the two samples showed the appearance of a peak with a similar mass/charge ratio as DON, but different retention times. This was postulated to be the DON metabolite, 3-epi-DON. A reduction of DON was not observed with the samples contaminated with a higher concentration of DON (41 μg/mL). The observed differences in the modification of DON in the assay with DON as the sole carbon source and the assay using naturally contaminated sources of wheat could be attributed to the naturally contaminated sources of wheat cultures containing additional carbon sources for the microbes to utilize. He et al. [28] were able to produce 3-epi-DON with their strain of *Devosia* with different carbon sources such as corn meal broth and a mixture of yeast and glucose. 3-Epi-DON may have been produced from our naturally contaminated sources of wheat, since 3-keto-DON may be further reduced to produce 3-epi-DON [29].

Our work extends previous studies that have demonstrated the potential to use mixed cultures of microbes to detoxify DON. Future work to assess how microbial assemblages change before, during, and after screening with DON will highlight what microorganisms are selected for under the pressure of DON. Additional work needs to be done to culture specific microorganisms that are unable to grow under the test conditions to greatly increase the probability of identifying an organism that can detoxify DON (e.g., the use of the iChip to identify the new antibiotic allowing scientists to screen for new microbes that are difficult to culture or unculturable with traditional laboratory practices) [30]. However, the transformation of DON to 3-keto-DON and to 3-epi-DON with our mixed cultures demonstrates the feasibility of our approach. Future work will aim to elucidate enzymes responsible for modification of DON as Chang et al. did by modifying ochratoxin A using a carboxypeptidase enzyme from the species *Bacillus amyloliquefaciens* ASAG1 [31]. Engineering yeast that express DON-detoxifying enzymes and/or adding purified enzymes that can convert DON to less toxic by-products will be of value in the fuel ethanol industry, where such strategies could reduce mycotoxins in fuel ethanol by-products destined for feed and food [27].

## 4. Materials and Methods

### 4.1. Field Collections

Plant and soil samples were collected at Virginia Tech’s Kentland Farm in Blacksburg VA, USA, on 13 September 2013. Microbial samples were collected from fresh leaves or plant debris, and from soil samples collected with a soil corer (10 × 1 in. diameter galvanized steel soil sampler, Zoro). Eleven samples were collected from six different collection sites including a field of oat (*Avena sativa*), a field of corn (*Zea mays)*, a landscape plot, a vineyard (*Vitis vinifera*), a peach orchard (*Prunus persica*), and an apple orchard (*Malus domestica*).

### 4.2. Selection of Microbes in the Presence of High Concentrations of DON

Plant samples were ground into a fine powder using a coffee grinder (Hamilton Beach, Model 80365, Southern Pines, NC, USA). Soil samples were placed in one-gallon zip lock bags and mixed thoroughly to displace soil clumps. Aliquots of 0.1 g of each sample were suspended in 1 mL of mineral salt medium (MM) [32] containing 100 μg/mL of DON as the sole carbon source. A negative control included 1 mL MM and 100 μg/mL DON without any environmental samples. Cultures were incubated on a shaker (New Brunswick Scientific Excella E-24 Incubator Shaker, Edison, NJ, USA) for 7 days at 120 RPM and 28 °C. After 7 days, 10 μL of each culture was added to 1 mL of MM and 100 μg/mL of DON and incubated for another 7 days under the same conditions. This process of subculturing and incubation was repeated four additional times for a total of six weeks.

Resulting cultures were screened for the disappearance of DON using gas chromatography/mass spectrometry (GC/MS) following standard protocols [33]. Each sample was diluted by adding 100 μL of culture to 1.9 mL of sterile water before GC/MS analysis; 250 μL of the dilution was added to 1.7 mL of acetonitrile and filtered through Whatman 1 qualitative paper. A 1 mL portion of the flow through was dried down in a glass tube using compressed air in a nitrogen evaporator set at 55 °C. Dried samples were then derivatized at room temperature with a mixture of 99 μL of N-trimethylsilylimidazole (TMSI) and 1 μL of trimethylchlorosilane (TMCS) for 20 min. Then, 500 μL of isooctane containing 0.5 μg/g of mirex (Sigma-Aldrich, St. Louis, MO, USA) was added to the glass tube and immediately vortexed, followed by an addition of 500 μL of water to quench the reaction. From the top organic layer, 150 μL was transferred to chromatography vials for GC/MS analysis.

An Agilent 6890/5975 system was used for GC/MS analysis operating in selected ion monitoring (SIM) mode. An autosampler in splitless mode injected 1 μL of each sample onto an HP-5MS column (0.25 mm inner-diameter, 0.25 μm film thickness, 30 m length) to detect DON. The inlet temperature was set at 280 °C with a column flow rate of 1.2 mL/min using helium. The initial column temperature was held at 150 °C for 1 min, increased to 280 °C at a rate of 30 °C/min, and held constant for 3.5 min. A post-run of 325 °C for 2.5 min was used to clean the column. DON was detected in SIM mode at a mass/charge ratio of 512.3 and had reference ions at 422.4 and 497.3. Mirex (hexachloropentadiene dimer) was used as an internal standard to check the quantitative precision of the instrument and was detected in SIM mode at a mass/charge ratio of 271.8 and had a reference ion of 275.8 [34]. A linear regression model was used to quantify DON with standards (Romer Labs, Austria and Sigma-Aldrich, St. Louis, MO, USA) at concentrations of 0.05, 0.10, 0.25, 0.5, 1.0, 2.50, and 5.0 μg/mL. Mycotoxin values were quantitated using a standard curve ranging from 0.05 to 1.0 μg/mL. Values determined to be greater than 1.0 μg/mL were quantitated using a curve that included the 2.5 and 5.0 μg/mL standards. The LOQ for the method was determined to be 0.2 μg/mL, based on standard protocols [33]. All cultures showing decreased levels of DON were transferred to 25% glycerol and stored at −80 °C.

Each culture that demonstrated decreased levels of DON were then further analyzed 4X (quadruplicate) to show consistency of decreased DON in culture assays with 100 μg/mL DON. A 50 μL sample of the glycerol stocks mentioned above were cultured into four separate tubes of R2A (Reasoner’s 2A; a medium for culturing slow-growing microorganisms; Sigma-Aldrich) liquid media for 2 days at 28 °C. After incubation, 100 μL of each culture was then added to a new culture tube containing 1 mL MM and 100 μg/mL DON and allowed to incubate with shaking at 120 RPM at 28 °C for 7 days. Mycotoxin extraction and GC/MS analysis described above was used to determine the amount of DON in each sample. All samples were made into a 25% glycerol stock and stored at −80 °C for future identification of microbes present.

### 4.3. Isolation of Individual DON Modifying Microbes

Mixed cultures that resulted in decreased levels of DON were selected and 200 μL of each culture was plated on solid R2A media and incubated for 7 days at 28 °C. After incubation, bacterial and fungal colonies of different morphologies were randomly selected and cultured in 1 mL of MM and 100 μg/mL DON for 7 days with shaking at 120 RPM at 28 °C. GC/MS preparation and analysis as described above was used to determine the concentration of DON in each sample after incubation. All pure cultures that demonstrated decreased levels of DON were made into a 25% glycerol stock and stored at −80 °C. Individual microbes that demonstrated decreased levels of DON were sequenced using 16S primers (27F and 518R bacterial 16S ribosomal primers) at the Biocomplexity Institute at Virginia Tech (Blacksburg, VA, USA).

### 4.4. Identification of Mixed Cultures Using 16S Ribosomal Sequencing

To assist in the identification of the microorganisms present in mixed cultures, 100 μL of frozen stock from the mineral media cultures with 100 μg/mL DON was incubated in 2 mL of R2A liquid media for 2 days at 28 °C. DNA from the mixed cultures was purified using a Thermo Scientific KingFisher mL nucleic acid purification machine (Thermo Scientific, Waltham, MA, USA) and Qiagen Puregene Yeast/Bac Kit B (Qiagen, Germantown, MD, USA). Samples of 40 ng/μL suspended in water were sent to MR DNA Laboratory in Shallowwater, Texas, for a diversity assay with 16S sequencing using the 27F primer. Illumina sequencing technology was used to generate an average of 20 K reads.

### 4.5. Thin Layer Chromatography to Identify DON Derivatives

Thin layer chromatography (TLC) was used to detect new DON products in mixed culture extracts (GC/MS analysis of TMS-derivatized samples run in SIM mode was used to measure the disappearance of DON). Samples were dried down using compressed air in the fume hood, and 200 μL of acetonitrile was then added and vortexed to ensure the DON derivatives were dissolved. Each sample was spotted 1 in. from the bottom of a 20 × 20 cm silica gel plate (60-F254, Millipore, Darmstadt, Germany). The plate was placed in a TLC tank (L × H × W 27.10 cm × 26.5 cm × 7.10 cm) using 48:92:10 hexane/ethyl acetate/methanol as the solvent. The solvent was allowed to run to approximately 3 cm away from the top of the plate. The plate was dried in a fume hood, then sprayed with NBP (nitrobenzylpyridine), heated for 30 min at 100 °C, and then lightly sprayed with TEPA (tetraethylenepentamine). Products that contained an epoxide group were stained blue [35].

### 4.6. Nuclear Magnetic Resonance to Identify Structure of DON Derivatives

In order to obtain sufficient product for nuclear magnetic resonance (NMR) analysis, samples were assayed in 10 mL of MM containing 100 μg/mL DON to produce enough by-product, incubated for one week with shaking at 28 °C, and were subsequently dried down using air. The residue was dissolved in 200 μL of acetonitrile and streaked on TLC plates as described above. Bands were visualized under UV light, marked with a pencil, and scraped off using a razor blade and placed in a glass vial. Deuterated chloroform was added to each vial and vortexed. ^1^H NMR spectra were recorded on a Bruker Avance II (500 Mhz) equipped with a Prodigy cryoprobe. Chemical shifts were referenced to residual proton signal of the CDCl3 solvent. MNova 11 was used to analyze the ^1^H data.

### 4.7. DON Assays with Mixed Cultures with Naturally Contaminated Wheat

Two wheat (*Triticum aestivum*) samples naturally infected by *Fusarium graminearum* and containing different concentrations of DON were used: Sample #13v-193 (7.1 μg/mL DON) and Sample #13w-7 (41.0 μg/mL DON). GC/MS methods were used to determine the concentrations of DON present in the samples. Samples were ground to a fine powder using a Stein mill (Steinlite Corp., Atchison, KS, USA).

To prepare for the assays, 50 μL of the mixed cultures were added to 2 mL of R2A liquid media and allowed to incubate for 2 days at 120 RPM and 28 °C. A 1.0 g sub-sample of each wheat sample was added to a 125 mL flask topped with a foam stopper (21–26 mm) and autoclaved on a dry cycle. Under sterile conditions, 4.5 mL of sterile water was added to each flask and 500 μL of each mixed culture was added to three flasks of each wheat sample, Sample #13v-193 and Sample #13w-7. Negative controls for each wheat sample were included without any mixed culture and only 5 mL of sterile water. All flasks were incubated for one week at 180 RPM and 28 °C. To analyze DON concentration after incubation, each sample was dried down in an oven set at 55 °C. Each 1.0 g sample was combined with 8 mL of an 84% (*v*/*v*) acetonitrile in DI water to extract DON, sonicated to release any clumps, then placed on a shaker at 200 RPM overnight at room temperature. The solvent was then cleaned by passing it through a column consisting of a 1:3 ratio of a 1.5 g mixture of C18 (40 um particle size) and aluminum oxide (active, neutral, 0.063 to 0.200 mm particle size range). An aliquot of 1 mL of the eluent was added to a glass test tube, dried and evaporated using a nitrogen evaporator set at 55 °C. Derivatization of samples was performed as described above and GC/MS analysis was performed operating in scan mode analyzing from 5.7 to 8.8 min; all other parameters of the GC/MS method was kept the same as described above.

## Figures and Tables

**Table 1:** DON modification by soil mixed cultures. Four sample replicates from Mixed Culture 1, 2, and 3 following incubation with 5 μg/mL DON.

Culture Sample	Replicate	DON (μg/mL) Analytical Rep 1	DON (μg/mL) Analytical Rep 2	Mean DON (μg/mL)
Mixed Culture 1-R1	1	0.16	0.16	0.16
Mixed Culture 1-R2	2	2.48	2.72	2.6
Mixed Culture 1-R3	3	3.12	3.04	3.08
Mixed Culture 1-R4	4	2.72	2.68	2.7
Mixed Culture 2-R1	1	<0.2	<0.2	<0.2
Mixed Culture 2-R2	2	4.32	3.8	4.06
Mixed Culture 2-R3	3	<0.2	<0.2	<0.2
Mixed Culture 2-R4	4	<0.2	<0.2	<0.2
Mixed Culture 3-R1	1	3.92	3.8	3.86
Mixed Culture 3-R2	2	3.92	3.76	3.84
Mixed Culture 3-R3	3	4.08	3.8	3.94
Mixed Culture 3-R4	4	3.28	3.64	3.46
Control-R1	1	4.46	4.48	4.47
Control-R2	2	4.48	4.28	4.38
Control-R3	3	3.84	4.04	3.94

**Table 2:** Grain culture extracts from naturally contaminated Wheat Sample #13v-193 (7.1 μg/mL DON) incubated with Mixed Cultures 1 and 2 were analyzed using GC/MS. Two separate assays were performed at different times with three replicates for Mixed Cultures 1 and 2 and the control. Sample ID 2 from Mixed Culture 1 in the first assay using Wheat Sample #13v-193 showed significant DON reduction compared to the negative control (below the limit of quantification, which is 0.20 μg/mL).

Sample ID	Culture ID	Assay	Replicate	Starting DON (μg/mL) in Wheat	Final DON (μg/mL) in Wheat
1	Mixed Culture 1	1	1	7.10	5.08
2	Mixed Culture 1	1	2	7.10	<0.20
3	Mixed Culture 1	1	3	7.10	0.08
4	Mixed Culture 1	2	1	7.10	7.04
5	Mixed Culture 1	2	2	7.10	7.76
6	Mixed Culture 1	2	3	7.10	5.88
					**4.3 (mean)**
7	Mixed Culture 2	1	1	7.10	4.0
8	Mixed Culture 2	1	2	7.10	6.4
9	Mixed Culture 2	1	3	7.10	3.48
10	Mixed Culture 2	2	1	7.10	7.56
11	Mixed Culture 2	2	2	7.10	7.88
12	Mixed Culture 2	2	3	7.10	7.28
					**6.1 (mean)**
Control	Control (no cultures)				**7.10 (mean)**

## Supplementary Materials

The following are available online at www.mdpi.com/2072-6651/9/4/141/s1, Table S1: Mixed and pure microbial samples, derived from either soil or plant material, that initially eliminated DON from cultures containing mineral media and 100 µg/mL of DON as the sole carbon source (GC/MS analysis indicated a value below the limit of quantification). Not all cultures were consistent at modifying DON in repeated assays. The percent of time each culture eliminated DON from the culture medium was based on how many times each culture eliminated DON from the culture over how many times each culture was assayed. Both mixed culture 1 and mixed culture 2 will modify DON in culture material 77% of the time they are assayed, Figure S1: GC/MS chromatogram of mixed culture 2 (7.7 min; detected in SIM mode with a target ion with a mass:charge ratio of 438.2 and reference ions at 318.2 and 303.1) after incubation in mineral media with 100 µg/mL of DON as the sole carbon source. DON is represented by the peak at 6.1 min. The DON incubated with mixed culture 2 was below the limit of detection of 0.20 µg/mL. Table S2: Proton data collected with a Bruker, Avance II, 500 MHz NMR for mixed culture 1 and DON. Proton data for 3-keto-DON was produced by Shima et al. [19]. Comparison of proton data for mixed culture 1 products with DON and 3-keto-DON confirm that mixed culture 1 contained 3-keto-DON. Proton data for mixed culture 2 was similar to mixed culture 1 (data not reported).
