# Supplementary material for: Modification of the Mycotoxin Deoxynivalenol Using Microorganisms Isolated from Environmental Samples
Source: Toxins (Basel). 2017 Apr 15;9(4):141. doi: 10.3390/toxins9040141 (PMC5408215; doi:10.3390/toxins9040141)
Supplement: Supplementary file 1 [file toxins-09-00141-s001.docx]

Supplementary Materials: Modification of the Mycotoxin Deoxynivalenol Using Microorganisms Isolated from Environmental Samples

Nina Wilson, Nicole McMaster, Dash Gantulga, Cara Soyars, Susan P. Mccormick, Ken Knott, Ryan S. Senger and David Schmale

**Table S1.** Mixed and pure microbial samples, derived from either soil or plant material, that initially eliminated DON from cultures containing mineral media and 100 µg/mL of DON as the sole carbon source (GC/MS analysis indicated a value below the limit of quantification). Not all cultures were consistent at modifying DON in repeated assays. The percent of time each culture eliminated DON from the culture medium was based on how many times each culture eliminated DON from the culture over how many times each culture was assayed. Both mixed culture 1 and mixed culture 2 will modify DON in culture material 77% of the time they are assayed.

| **Sample** | **Collection Area** | **Mixed/ Pure Culture** | **Collection Method** | **% Time DON Eliminated from Culture** | **DON (µg/mL), Initial Screen** | |
| --- | --- | --- | --- | --- | --- | --- |
|  |  |  |  |  | **Rep 1** | **Rep 2** |
| Mixed culture 1 | Soil- Landscape Plot | Mixed | Soil Corer | 77 | <0.20 | <0.20 |
| Mixed culture 2 | Soil- Landscape Plot | Mixed | Soil Corer | 77 | <0.20 | <0.20 |
| Mixed culture 3 | Soil- Landscape Plot | Mixed | Soil Corer | 37 | <0.20 | <0.20 |
| Pure culture 1 | Plant- Small Grain Field | Pure - *Achromobacter* | Leaf material & debris | 33 | <0.20 | <0.20 |
| Pure culture 2 | Soil- Landscape Plot | Pure – *Pseudomonas* | Soil Corer | 50 | <0.20 | <0.20 |


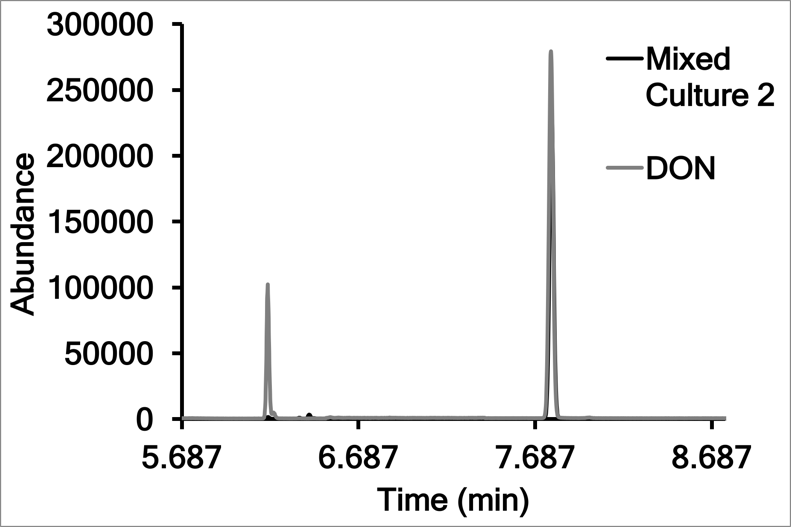


**Figure S1.** GC/MS chromatogram of mixed culture 2 (7.7 min; detected in SIM mode with a target ion with a mass:charge ratio of 438.2 and reference ions at 318.2 and 303.1) after incubation in mineral media with 100 µg/mL of DON as the sole carbon source. DON is represented by the peak at 6.1 min. The DON incubated with mixed culture 2 was below the limit of detection of 0.20 µg/mL.

| **Position** | **Mixed culture 1** | **DON** | **3-keto-DON** |
| --- | --- | --- | --- |
| 1 |  |  |  |
| 2 | 3.52 (s) | 3.65 (d, 4.48) | 3.52 (s) |
| 3 |  | 4.56 (td, 4.55, 11) |  |
| 4 | 3.14 (d, 19.26),  2.27 (br d, 19.30) | 2.23 (dd, 14.63, 4.07),  2.11 (dd, 15, 11) | 3.13 (d, 19),  2.28 (br d, 19) |
| 5 |  |  |  |
| 6 |  |  |  |
| 7 | 4.91 (br s) | 4,86 (br s) | 4.90 (br s) |
| 8 |  |  |  |
| 9 |  |  |  |
| 10 | 6.54 (br d, 5.92) | 6.63 (br d, 6.02) | 6.55 (br d, 6) |
| 11 | 4.58 (br d, 5.93) | 4. 83 (d, 5.89) | 4.57 (br d, 6) |
| 12 |  |  |  |
| 13 | 3.35 (d, 4.24),  3.23 (d, 4.21) | 3.18 (d, 4.31),  3.09 (d, 4) | 3.35 (d, 4),  3.22 (d, 4) |
| 14 | 1.33 (3H, s) | 1.16 (3H, s) | 1.33 (3H, s) |
| 15 | 3.90 (d, 11.72),  3.73 (d, 12.07) | 3.92 (d, 12.25),  3.76 (d, 12.25) | 3.90 (d, 12),  3.74 (d, 12) |
| 16 | 1.90 (3H, b s) | 1.91 (3H, br s) | 1.90 (3H, br s) |

**Table S2.** Proton data collected with a Bruker, Avance II, 500 MHz NMR for mixed culture 1 and DON. Proton data for 3-keto-DON was produced by Shima et al. [19]. Comparison of proton data for mixed culture 1 products with DON and 3-keto-DON confirm that mixed culture 1 contained 3-keto-DON. Proton data for mixed culture 2 was similar to mixed culture 1 (data not reported).
